# Immunogenicity and Safety of Investigational MenABCWY Vaccine and of 4CMenB and MenACWY Vaccines Administered Concomitantly or Alone: a Phase 2 Randomized Study of Adolescents and Young Adults

**DOI:** 10.1128/mSphere.00553-21

**Published:** 2021-11-17

**Authors:** Jiří Beran, Daniel Dražan, Igwebuike Enweonye, Chiranjiwi Bhusal, Daniela Toneatto

**Affiliations:** a Vaccination and Travel Medicine Center, Hradec Králové, Czech Republic; b Institute for Postgraduate Health Education, Prague, Czech Republic; c General practitioner, Jindřichův Hradec, Czech Republic; d GSK, Amsterdam, The Netherlands; e GSK, Siena, Italy; UTMB

**Keywords:** immunogenicity, investigational, MenABCWY, *Neisseria meningitidis*

## Abstract

This phase 2, randomized, open-label study assessed the immunogenicity and safety of an investigational meningococcal ABCWY vaccine (MenABCWY) that contains components of licensed vaccines against meningococcal serogroup B (4CMenB) and serogroups ACWY (MenACWY). A total of 500 healthy 10- to 25-year-old participants were randomly assigned to one of five study groups in a 1:1:1:1:1 ratio. Four groups received two doses 2 months apart of MenABCWY and 4CMenB plus MenACWY administered concomitantly in the same arm (4CMenB+ACWY/S group) or different arms (4CMenB+ACWY/D group) or 4CMenB administered alone. A fifth group received a single MenACWY dose. Immunogenicity was determined by serum bactericidal assay using human complement (hSBA). The study was powered to assess immunological interference against pooled serogroup B test strains. One month after the second vaccine dose, hSBA geometric mean titers (GMTs) (with 80% confidence intervals [CI]) against pooled serogroup B strains were 31.84 (80% CI, 28.18 to 35.98), 38.48 (80% CI, 34.23 to 43.26), 40.08 (80% CI, 35.44 to 45.33), and 42.38 (80% CI, 37.31 to 48.13) in the MenABCWY, 4CMenB+ACWY/S, 4CMenB+ACWY/D, and 4CMenB groups, respectively. Immune responses (GMTs and 80% CIs) were lower for PorA and NHBA serogroup B test strains in the MenABCWY group compared to the 4CMenB+ACWY/D group and 4CMenB group. Evaluation of solicited and unsolicited adverse events (AEs) identified no safety concerns for the MenABCWY vaccine. One serious AE (syncope in the 4CMenB group) was considered related to vaccination. In conclusion, there is no evidence of substantial immunological interference between 4CMenB and MenACWY vaccine components against serogroup B. The safety and tolerability profile of the investigational MenABCWY vaccine was acceptable. (This study has been registered at ClinicalTrials.gov under registration no. NCT03587207.)

**IMPORTANCE** The bacterial species Neisseria meningitidis is a major cause of meningitis, with six meningococcal groups (serogroups) causing most cases. A licensed vaccine, MenACWY (Menveo), targets four of these meningococcal serogroups, and another vaccine, 4CMenB (Bexsero), targets serogroup B. A combined vaccine (MenABCWY) that targets all five serogroups is under development to simplify the vaccination schedule. In a previous study, the immune response to serogroup B was found to be overall higher in individuals who received 4CMenB than in those who received an investigational MenABCWY vaccine. We investigated this further by giving healthy adolescents and young adults the MenABCWY vaccine, 4CMenB plus MenACWY vaccine in the same or different arms, 4CMenB vaccine alone, or MenACWY vaccine alone. Immunogenicity results for serogroup B across study groups suggest no major interference between the MenB and MenACWY vaccine components. This supports further development of the combined MenABCWY vaccine.

## INTRODUCTION

Neisseria meningitidis is a leading cause of bacterial meningitis and sepsis in children and young adults, with six meningococcal serogroups (A, B, C, W, X, and Y) accounting for the majority of cases of invasive meningococcal disease (IMD) worldwide (1). A quadrivalent meningococcal conjugate vaccine using a nontoxic diphtheria cross-reacting mutant (CRM_197_) as carrier protein, MenACWY-CRM (Menveo, GSK), was first licensed in 2010. Effectiveness data show this vaccine provides broad protection against IMD caused by serogroups A, C, W, and Y in all age groups (2). The four-component meningococcal serogroup B (MenB) vaccine 4CMenB (Bexsero, GSK) was first licensed in 2013 (3). Postlicensure experience shows 4CMenB is highly effective in preventing invasive MenB disease in children and young adults (3–5).

An investigational vaccine (MenABCWY) combining MenACWY and 4CMenB antigens is being developed to provide protection against IMD caused by five serogroups to simplify the vaccination schedule and potentially improve vaccine coverage in the population. The MenABCWY vaccine has been shown to be immunogenic, with an acceptable safety profile in adolescents (6, 7) and 10- to 25-year-old individuals (8). In one phase 2 randomized study, immune responses against serogroup B test strains were substantial in participants who received the MenABCWY vaccine in a two-dose schedule, although responses were overall higher in those who received 4CMenB alone (8). The reason for this observation is unknown, and multiple factors might have contributed, which may include immune interference, possibly due to immunological stress to lymph nodes in the arm where the combined vaccine is administered, or physicochemical interactions between vaccine components (8–10).

This phase 2 randomized study (ClinicalTrials.gov registration no. NCT03587207) was conducted to assess if there is immune interference when two doses of the MenABCWY vaccine are administered 2 months apart. Healthy 10- to 25-year-old individuals were administered MenABCWY vaccine, 4CMenB plus MenACWY vaccines administered concomitantly in the same arm (4CMenB+ACWY/S group) or in different arms (4CMenB+ACWY/D group), 4CMenB vaccine alone, or MenACWY vaccine alone.

## RESULTS

### Study population.

**FIG 1:**
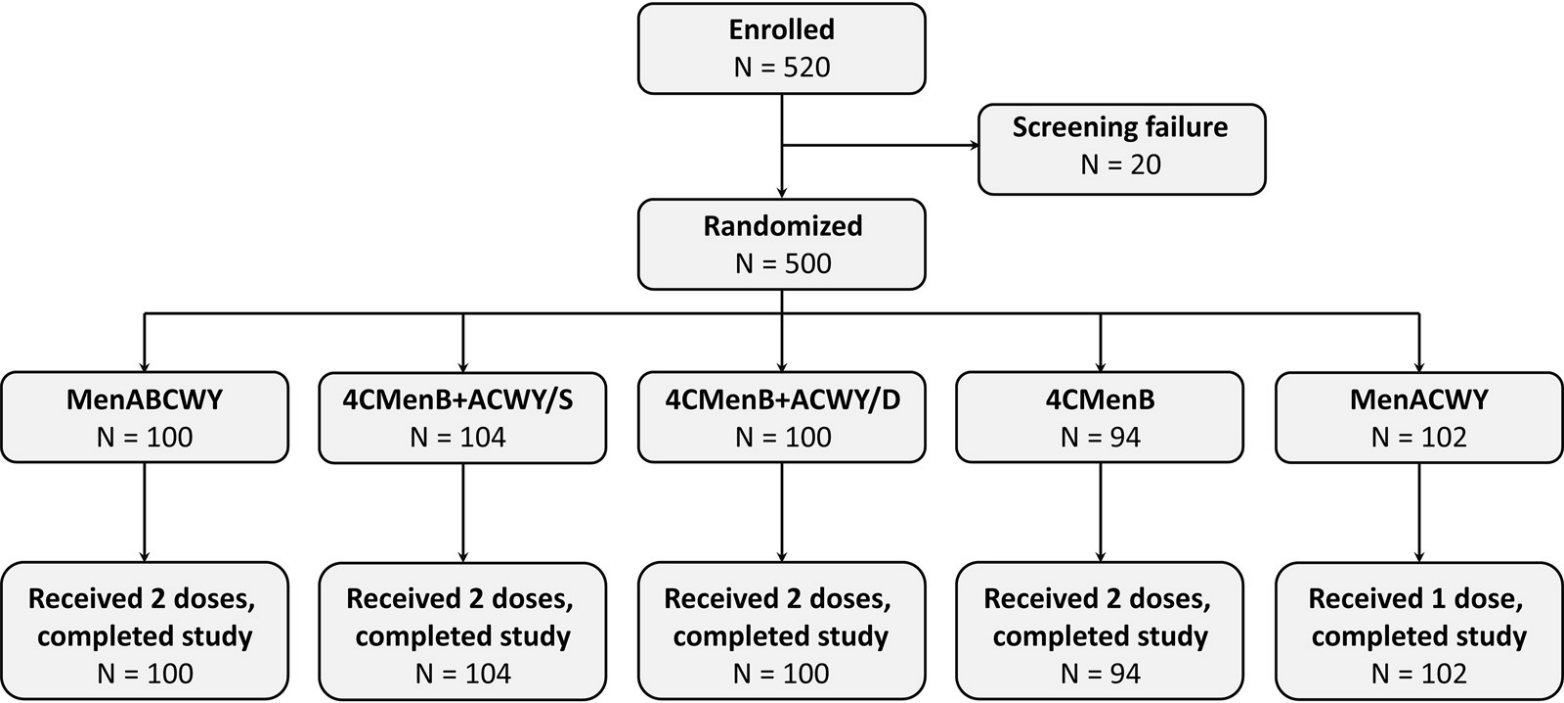
Disposition of the study participants. All groups, apart from MenACWY, received vaccination on study days 1 and 61, with a 30-day follow-up period. The MenACWY group received vaccination on day 1, with a 30-day follow-up period. N, number of participants; /S, vaccines administered concomitantly in the same arm; /D, vaccines administered concomitantly in different arms.

A total of 520 participants 10 to 25 years of age were enrolled, of which 500 met the eligibility criteria and were randomly assigned to one of the five study groups (Fig. 1). Of the 500 randomized subjects, 100 received two doses of MenABCWY (MenABCWY group), 104 received two doses of 4CMenB plus MenACWY administered concomitantly in the same arm (4CMenB+ACWY/S group), 100 received two doses of 4CMenB plus MenACWY administered in different arms (4CMenB+ACWY/D group), and 94 received two doses of 4CMenB (4CMenB group). In the fifth group, 102 subjects received a single dose of MenACWY, according to the schedule approved in adolescents and adults. All randomized participants received each scheduled vaccine dose and completed the study. Age and gender percentages were well balanced among study groups (Table 1). All participants were white.

**TABLE 1:** Demographic characteristics of the study participants^*a*^

Characteristic	Result for vaccine group				
	MenABCWY (*n* = 100)	4CMenB+MenACWY/S (*n* = 104)	4CMenB+MenACWY/D (*n* = 100)	4CMenB (*n* = 94)	MenACWY (*n* = 102)
Age (yr), mean ± SD	17.1 ± 4.34	16.9 ± 4.28	17.1 ± 4.49	17.4 ± 4.64	17.1 ± 4.57

Age range, no. (%)					
10–17 yr	53 (53.0)	50 (48.1)	48 (48.0)	49 (52.1)	50 (49.0)
18–25 yr	47 (47.0)	54 (51.9)	52 (52.0)	45 (47.9)	52 (51.0)

Male, no. (%)	53 (53.0)	56 (53.8)	51 (51.0)	41 (43.6)	53 (52.0)

a All participants were randomized. SD, standard deviation; /S, vaccines administered concomitantly in the same arm; /D, vaccines administered concomitantly in different arms; *n*, number of participants; no., number of participants in a specific category; yr, years.

### Immunogenicity.

**FIG 2:**
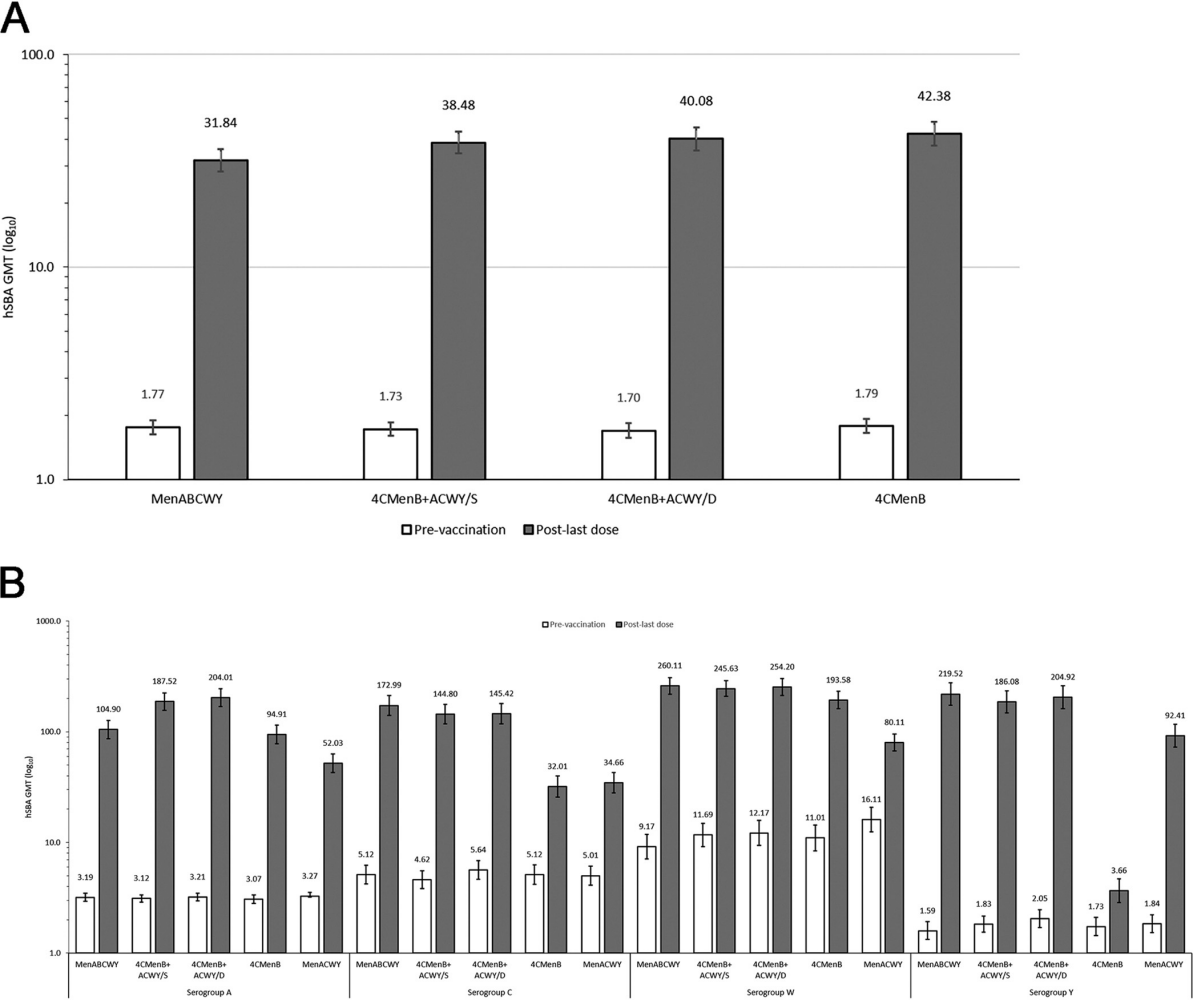
hSBA GMTs (80% CI) against (A) pooled serogroup B test strains and (B) serogroups A, C, W, and Y at prevaccination and 1 month post-last vaccination (per-protocol population for immunogenicity). (A) Pooled serogroup B test strains. (B) Serogroups A, C, W, and Y. CI, confidence interval; GMT, geometric mean titer; hSBA, serum bactericidal assay with human complement; /S, vaccines administered concomitantly in the same arm; /D, vaccines administered concomitantly in different arms.

**FIG 3:**
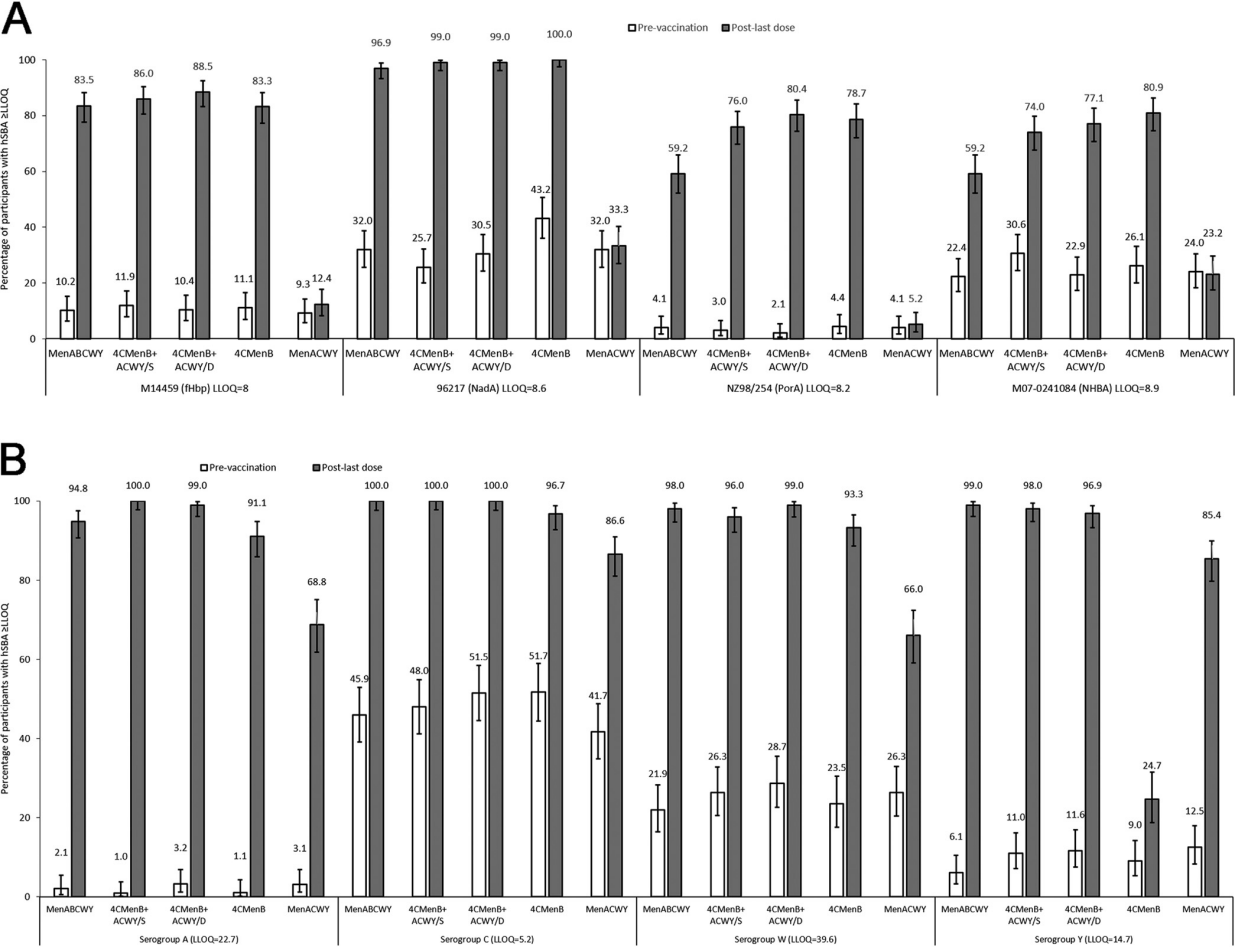
Percentage of participants (80% CI) with an hSBA titer that is ≥lower limit of quantitation (LLOQ) against (A) serogroup B test strains and (B) serogroups A, C, W, and Y at prevaccination and 1 month post-last vaccination (per-protocol population for immunogenicity). (A) Serogroup B test strains. (B) Serogroups A, C, W, and Y. CI, confidence interval; hSBA, serum bactericidal assay with human complement; /S, vaccines administered concomitantly in same arm; /D, vaccines administered concomitantly in different arms.

The primary endpoint was the immune response against pooled serogroup B test strains (for antigens fHbp, NadA, PorA, and NHBA) and serogroups A, C, W, and Y 1 month after the last vaccination. This was measured by serum bactericidal assay using human complement (hSBA) (Fig. 2; see Fig. S1 in the supplemental material), the percentage of participants with an hSBA titer that is greater than or equals the lower limit of quantitation (≥LLOQ) (Fig. 3) or with a 4-fold increase in hSBA titer (see Fig. S2 the supplemental material), and by geometric mean ratio (GMR) of hSBA titers (see Table S1 in the supplemental material).

10.1128/mSphere.00553-21.1 FIG S1 hSBA GMTs (80% CI) against (A) pooled serogroup B test strains, (B) each serogroup B test strain, and (C) serogroups A, C, W, and Y at prevaccination and 1 month post-first and post-last vaccinations (per-protocol population for immunogenicity). Note that prevaccination GMTs for the post-first-dose cohort (participants with assay results at day 31, except MenACWY group) differ slightly from those for post-last-dose cohort (participants with assay results at day 91 or, for the MenACWY group only, day 31) because of different numbers of participants with assay results. Prevaccination GMTs for the post-last-dose cohort are shown. CI, confidence interval; GMT, geometric mean titer; hSBA, serum bactericidal assay with human complement; /S, vaccines administered concomitantly in the same arm; /D, vaccines administered concomitantly in different arms. Download FIG S1, DOCX file, 0.2 MB. Copyright © 2021 Beran et al. 2021 Beran et al. https://creativecommons.org/licenses/by/4.0/ This content is distributed under the terms of the Creative Commons Attribution 4.0 International license.

10.1128/mSphere.00553-21.2 FIG S2 Percentage of participants (80% CI) with 4-fold increase in hSBA titer from baseline to 1 month post-first and post-last vaccinations (per-protocol population for immunogenicity). (A) Serogroup B test strains; (B) serogroups A, C, W, and Y. CI, confidence interval; hSBA, serum bactericidal assay with human complement; /S, vaccines administered concomitantly in the same arm; /D, vaccines administered concomitantly in different arms. Download FIG S2, DOCX file, 0.1 MB. Copyright © 2021 Beran et al. 2021 Beran et al. https://creativecommons.org/licenses/by/4.0/ This content is distributed under the terms of the Creative Commons Attribution 4.0 International license.

10.1128/mSphere.00553-21.4 TABLE S1 Geometric mean ratio of postvaccination to baseline (prevaccination) geometric mean titers 1 month post-last vaccination (per-protocol population for immunogenicity). CI, confidence interval; N, number of participants who received vaccination and had assay results available; /S, vaccines administered concomitantly in the same arm; /D, vaccines administered concomitantly in different arms. Download Table S1, DOCX file, 0.01 MB. Copyright © 2021 Beran et al. 2021 Beran et al. https://creativecommons.org/licenses/by/4.0/ This content is distributed under the terms of the Creative Commons Attribution 4.0 International license.

The study was powered to assess only immunological interferences against pooled serogroup B test strains 1 month after the last vaccination. This analysis showed postvaccination geometric mean titers (GMTs) against pooled serogroup B strains in the 4CMenB+ACWY/S group were similar to those in the 4CMenB+ACWY/D group (Fig. 2). The ratio of GMTs (4CMenB+ACWY/S versus 4CMenB+ACWY/D) was 0.96 (80% confidence interval [CI], 0.83 to 1.10), indicating no statistically significant difference. The ratio of MenABCWY versus 4CMenB+ACWY/S GMTs was 0.83 (80% CI, 0.72 to 0.95) against pooled serogroup B strains.

For serogroups A, C, W, and Y, GMTs after the second dose were similar between the 4CMenB+ACWY groups and MenABCWY group, apart from GMTs for serogroup A, which were lower in the MenABCWY group (104.90; 80% CI, 87.02 to 126.47) than in the 4CMenB+ACWY/S (187.52; 80% CI, 156.94 to 224.05) and 4CMenB+ACWY/D (204.01; 80% CI, 169.06 to 246.18) groups (Fig. 2). Similar trends were found in GMRs of postvaccination to baseline GMTs (Table S1). One month after the single dose of MenACWY, GMTs against serogroups A, C, W, and Y were lower than after two doses of MenABCWY or 4CMenB+MenACWY (Fig. 2; Table S1).

The postvaccination GMTs against pooled serogroup B strains were lower in the MenABCWY group (31.84; 80% CI, 28.18 to 35.98) than in the 4CMenB group (42.38; 80% CI, 37.31 to 48.13) (Fig. 2). GMTs against individual serogroup B test strains after the second dose were similar between the two 4CMenB+ACWY groups (Fig. S1), as were GMRs of postvaccination to baseline GMTs (Table S1). Immune responses against serogroup B test strains for NadA, PorA, and NHBA antigens were lower in the MenABCWY group than in the 4CMenB group and were also lower in the MenABCWY group than in the 4CMenB+ACWY/D group for PorA and NHBA (Fig. S1 and Table S1).

The percentages of participants with hSBA titers that are ≥LLOQ against each serogroup B test strain and serogroups A, C, W, and Y were similar between the two 4CMenB+ACWY groups 1 month after the second vaccine dose (Fig. 3). The percentage was lower in the MenABCWY group than in the 4CMenB+ACWY/S group for serogroup A (94.8% versus 100%). The percentage was also lower in the MenABCWY group than in the 4CMenB+ACWY/S and 4CMenB+ACWY/D groups for serogroup B test strains for PorA (59.2% versus 76.0 and 80.4%) and NHBA antigens (59.2% versus 74.0 and 77.1%). For the remaining serogroups and strains, the percentages of participants with hSBA titers that were ≥LLOQ after two doses were similar among the three groups (Fig. 3).

The percentages of participants with a 4-fold increase in hSBA titer against each serogroup B strain and serogroups A, C, W, and Y were similar in the two 4CMenB+ACWY groups after two doses (Fig. S2). In the MenABCWY group, the percentages of participants with a 4-fold increase were either lower than (NHBA and serogroup A) or similar to (the remaining strains and serogroups) those of the 4CMenB+ACWY groups (Fig. S2). In the 4CMenB group, the percentages of participants with hSBA titers that were ≥LLOQ or a 4-fold increase in hSBA titer for the serogroup B test strains were generally similar to those in the 4CMenB+ACWY groups (Fig. 3; Fig. S2).

Immune response results 1 month post-first vaccination, which were secondary study endpoints, are shown in Fig. S1 to S3 in the supplemental material for the groups that received two vaccine doses.

10.1128/mSphere.00553-21.3 FIG S3 Percentage of participants (80% CI) with hSBA titer of ≥lower limit of quantitation at prevaccination and 1 month post-first and post-last vaccinations (per-protocol population for immunogenicity). Note that prevaccination GMTs for the post-first-dose cohort (participants with assay results at day 31, except for the MenACWY group) differ slightly from those for the post-last-dose cohort (participants with assay results at day 91 or, for the MenACWY group only, day 31) because of different numbers of participants with assay results. Prevaccination GMTs for the post-last-dose cohort are shown. (A) Serogroup B test strains; (B) serogroups A, C, W, and Y. CI, confidence interval; GMT, geometric mean titer; hSBA, serum bactericidal assay with human complement; LLOQ, lower limit of quantitation; /S, vaccines administered concomitantly in the same arm; /D, vaccines administered concomitantly in different arms. Download FIG S3, DOCX file, 0.2 MB. Copyright © 2021 Beran et al. 2021 Beran et al. https://creativecommons.org/licenses/by/4.0/ This content is distributed under the terms of the Creative Commons Attribution 4.0 International license.

### Reactogenicity and safety.

At least one solicited local adverse event (AE) was reported in the 7 days following vaccination by 90.0 to 97.1% of participants after the first dose and 89.0 to 96.0% after the second dose of MenABCWY, 4CMenB+ACWY/S, 4CMenB+ACWY/D, or 4CMenB and 57.8% of those receiving the single dose of MenACWY (Table 2). The most frequent solicited local AE in all groups was pain, which was reported by 89.0 to 96.2% of participants following the first dose and 87.0 to 95.0% after the second dose of MenABCWY, 4CMenB+ACWY/S, 4CMenB+ACWY/D, or 4CMenB and 51.0% of participants receiving MenACWY (Table 3). Severe pain was reported by 15.0, 21.2, 16.0, and 12.8% of participants after the first dose of MenABCWY, 4CMenB+ACWY/S, 4CMenB+ACWY/D, and 4CMenB, respectively, and by 19.0, 21.2, 26.0, and 16.0%, respectively, after the second dose. Severe pain was reported by 2.9% of MenACWY recipients (Table 3).

**TABLE 2:** Numbers and percentage of participants reporting solicited adverse events (AEs) within 1 to 7 days of vaccination and unsolicited AEs during the 30-day postvaccination period^*a*^

AE	No. (%) of participants with AE^*b*^				
	MenABCWY (*n* = 100)	4CMenB+MenACWY/S (*n* = 104)	4CMenB+MenACWY/D (*n* = 100)	4CMenB (*n* = 94)	MenACWY (*n* = 102)
Solicited AE^*c*^					
Dose 1					
Any	93 (93.0)	102 (98.1)	97 (97.0)	93 (98.9)	76 (74.5)
Local	90 (90.0)	101 (97.1)	97 (97.0)	90 (95.7)	59 (57.8)
Systemic	69 (69.0)	73 (70.2)	66 (66.0)	61 (64.9)	60 (58.8)
Dose 2					
Any	90 (90.0)	98 (94.2)	96 (96.0)	91 (96.8)	
Local	89 (89.0)	98 (94.2)	96 (96.0)	90 (95.7)	
Systemic	71 (71.0)	65 (62.5)	69 (69.0)	63 (67.0)	

At least 1 unsolicited AE	23 (23.0)	27 (26.0)	26 (26.0)	23 (24.5)	15 (14.7)
Related to vaccination	8 (8.0)	11 (10.6)	13 (13.0)	11 (11.7)	4 (3.9)
Leading to medically attended visit	14 (14.0)	17 (16.3)	14 (14.0)	13 (13.8)	6 (5.9)

a Shown are the numbers and percentages of participants reporting solicited adverse events (AEs) within 1 to 7 days of vaccination and unsolicited AEs during the 30-day postvaccination period following one dose (MenACWY group) or one and two vaccine doses (all other groups). All participants were randomized.

b /S, vaccines administered concomitantly in the same arm; /D, vaccines administered concomitantly in different arms; *n*, number of participants; no., number of participants in a specific category.

c Solicited local AEs include erythema, swelling, induration, and pain, and solicited systemic AEs include arthralgia, fatigue, nausea, headache, myalgia, and fever.

**TABLE 3:** Numbers and percentages of participants reporting solicited local and systemic adverse events (AEs) within 1 to 7 days of each vaccine dose^*a*^

AE	Dose	Severity^*b*^	No. (%) of participants reporting AE^*c*^				
			MenABCWY (*n* = 100)	4CMenB+MenACWY/S (*n* = 104)	4CMenB+MenACWY/D (*n* = 100)	4CMenB (*n* = 94)	MenACWY (*n* = 102)
Local							
Erythema	1	Any	17 (17.0)	19 (18.3)	18 (18.0)	9 (9.6)	7 (6.9)
		>100 mm	3 (3.0)	2 (1.9)	1 (1.0)	0	1 (1.0)
	2	Any	15 (15.0)	18 (17.3)	15 (15.0)	10 (10.6)	
		>100 mm	1 (1.0)	1 (1.0)	2 (2.0)	0	
Swelling	1	Any	20 (20.0)	21 (20.2)	16 (16.0)	13 (13.8)	10 (9.8)
		>100 mm	2 (2.0)	1 (1.0)	1 (1.0)	0	0
	2	Any	13 (13.0)	14 (13.5)	15 (15.0)	12 (12.8)	
		>100 mm	1 (1.0)	1 (1.0)	1 (1.0)	0	
Induration	1	Any	9 (9.0)	15 (14.4)	12 (12.0)	6 (6.4)	4 (3.9)
		>100 mm	1 (1.0)	0	1 (1.0)	0	0
	2	Any	6 (6.0)	11 (10.6)	12 (12.0)	12 (12.8)	
		>100 mm	0	1 (1.0)	1 (1.0)	0	
Pain	1	Any	89 (89.0)	100 (96.2)	95 (95.0)	88 (93.6)	52 (51.0)
		Severe	15 (15.0)	22 (21.2)	16 (16.0)	12 (12.8)	3 (2.9)
	2	Any	87 (87.0)	97 (93.3)	95 (95.0)	87 (92.6)	
		Severe	19 (19.0)	22 (21.2)	26 (26.0)	15 (16.0)	

Systemic							
Arthralgia	1	Any	20 (20.4)	15 (15.0)	9 (9.4)	9 (10.5)	18 (18.4)
		Severe	2 (2.0)	2 (2.0)	1 (1.0)	0	0
	2	Any	22 (22.4)	19 (18.4)	12 (12.2)	17 (18.5)	
		Severe	5 (5.1)	1 (1.0)	1 (1.0)	1 (1.1)	
Fatigue	1	Any	56 (56.6)	61 (59.8)	55 (55.0)	46 (50.0)	50 (50.0)
		Severe	9 (9.1)	8 (7.8)	2 (2.0)	7 (7.6)	7 (7.0)
	2	Any	58 (58.0)	61 (58.7)	62 (62.0)	56 (59.6)	
		Severe	10 (10.0)	7 (6.7)	9 (9.0)	11 (11.7)	
Nausea	1	Any	16 (16.5)	23 (22.8)	14 (14.4)	13 (15.3)	14 (14.1)
		Severe	1 (1.0)	3 (3.0)	1 (1.0)	0	0
	2	Any	16 (16.3)	13 (12.7)	15 (15.3)	17 (18.3)	
		Severe	2 (2.0)	3 (2.9)	1 (1.0)	2 (2.2)	
Headache	1	Any	40 (40.8)	46 (45.1)	38 (38.8)	36 (39.1)	36 (36.4)
		Severe	4 (4.1)	4 (3.9)	3 (3.1)	4 (4.3)	4 (4.0)
	2	Any	52 (53.1)	36 (34.6)	37 (37.0)	39 (41.9)	
		Severe	6 (6.1)	5 (4.8)	3 (3.0)	2 (2.2)	
Myalgia	1	Any	34 (34.7)	29 (28.7)	27 (27.8)	21 (24.1)	28 (28.3)
		Severe	6 (6.1)	4 (4.0)	0	2 (2.3)	4 (4.0)
	2	Any	41 (41.8)	38 (36.9)	35 (35.4)	38 (40.4)	
		Severe	7 (7.1)	4 (3.9)	2 (2.0)	3 (3.2)	
Fever (≥38°C)	1	Yes	6 (6.0)	5 (4.9)	5 (5.0)	1 (1.1)	3 (3.0)
	2	Yes	6 (6.0)	3 (2.9)	3 (3.0)	1 (1.1)	

a All participants were randomized.

b Severe pain, arthralgia, fatigue, headache, and myalgia were defined as preventing normal daily activities; severe nausea was defined as leading to minimal or no oral intake; severe fever was defined as a body temperature of ≥40°C (no reports).

c /S, vaccines administered concomitantly in the same arm; /D, vaccines administered concomitantly in different arms; *n*, number of participants; no., number of participants in a specific category.

Any solicited systemic AE was reported by 64.9 to 70.2% of participants after the first dose and 62.5 to 71.0% after the second dose of MenABCWY, 4CMenB+ACWY/S, 4CMenB+ACWY/D, or 4CMenB and 58.8% of those receiving the single dose of MenACWY (Table 2). The most commonly reported solicited systemic AEs were fatigue (50.0 to 59.8% after the first dose and 58.0 to 62.0% after the second dose in the MenABCWY, 4CMenB+ACWY/S, 4CMenB+ACWY/D, or 4CMenB groups and 50.0% in the MenACWY group) and headache (38.8 to 45.1%, 34.6 to 53.1%, and 36.4%, respectively) (Table 3). Solicited systemic AEs were generally mild to moderate in severity. Severe fatigue was reported by 2.0 to 9.1% of participants after the first dose and 6.7 to 11.7% after the second dose of MenABCWY, 4CMenB+ACWY/S, 4CMenB+ACWY/D, or 4CMenB and 7.0% of those receiving MenACWY, and severe headache was reported by 3.1 to 4.3%, 2.2 to 6.1%, and 4.0%, respectively (Table 3). Body temperature of ≥38°C was reported by 1.1 to 6.0% of participants in each group after each dose, and there were no reports of fever of ≥40°C.

At least one unsolicited AE was reported during the 30-day postvaccination period by 23.0 to 26.0% of participants receiving MenABCWY, 4CMenB+ACWY/S, 4CMenB+ACWY/D, or 4CMenB and 14.7% of MenACWY recipients (Table 2). The most frequently reported unsolicited AEs were injection site pain, reported by 4.0, 2.9, 4.0, and 5.3% of participants receiving MenABCWY, 4CMenB+ACWY/S, 4CMenB+ACWY/D, and 4CMenB, respectively, and no MenACWY recipients, upper respiratory tract infection (5.0, 2.9, 0, 3.2, and 2.9%, respectively), and injection site induration (2.0, 4.8, 1.0, 4.3, and 0%, respectively).

At least one medically attended unsolicited AE was reported by 5.9 to 16.3% of participants in each group (Table 2), most commonly (maximum incidence, 2.9%) upper respiratory tract infection, tonsillitis, viral infection, pharyngitis, ligament sprain, concussion, toothache, and syncope. There were five serious AEs (SAEs) reported by five participants: concussion (two participants in the 4CMenB+ACWY/S group), bone fracture (one participant in the 4CMenB+ACWY/D group and one in the 4CMenB group), and syncope (one participant in the 4CMenB group). None were assessed as related to vaccination, apart from the SAE of syncope, which was considered possibly or probably related to vaccination. The syncope episode occurred 1 day post-last vaccination. The subject received intravenous physiologic solution and was discharged from the hospital the same day, with full recovery. There were no reports of the AE of special interest (arthritis), and no subject was withdrawn from the study due to an AE. No deaths were reported during the study.

## DISCUSSION

One of the main objectives of this study was to estimate the extent of potential immune interference between the different antigens when the investigational MenABCWY vaccine was administered to healthy adolescents and young adults. The study was powered only to assess immunological interferences against pooled serogroup B test strains; as explained in Materials and Methods, the 4CMenB+ACWY/S group was regarded as statistically inferior if the 2-sided 80% CIs of the ratio of the GMT with 4CMenB+ACWY/D as a control was lower than 1 at 1 month after the second vaccine dose. The immune response against individual vaccine antigens was analyzed descriptively.

Immune responses of participants given 4CMenB and MenACWY concomitantly in the same arm or in different arms suggest no substantial immunological interference between the vaccine antigens since GMTs against pooled serogroup B test strains were similar between these groups 1 month after two doses. Analysis of pooled serogroup B test strains showed the GMT ratio and 80% CI for MenABCWY versus 4CMenB+MenACWY/S were lower than 1, suggesting a trend for interference. Given the lack of substantial immunological interference between 4CMenB and MenACWY, this trend is likely to have another cause, which is unknown.

Other descriptive observations can be made from this study. For the individual serogroup B test strains, immune responses were similar between the two 4CMenB+ACWY groups. Immune responses against NadA, PorA, and NHBA MenB test strains were lower in the MenABCWY group than in the 4CMenB group and were lower for PorA and NHBA in the MenABCWY group than in subjects receiving 4CMenB+ACWY in different arms. Again, the reason for this observation is unknown, and the possibility that this was related to the descriptive nature of the analyses cannot be excluded.

Overall, immune responses following two doses of MenABCWY or 4CMenB+MenACWY were similar to those observed in previous studies of the same vaccination schedule in healthy adolescents and young adults (6–8). As expected, immune responses to serogroups A, C, W, and Y following one dose of MenACWY were lower than those following two doses of MenABCWY or 4CMenB+MenACWY.

A similar percentage of participants reported solicited local or systemic AEs in all groups administered two vaccine doses. The group that received a single dose of MenACWY vaccine had the lowest rates of solicited AEs. The most frequent solicited AEs were consistent with those reported in previous studies (6–8), and no concerning safety signals were identified for the investigational MenABCWY vaccine. Five SAEs were reported in five participants, of which one (syncope following 4CMenB) was considered related to vaccination.

In summary, administration of two doses of a combined MenABCWY vaccine to healthy individuals 10 to 25 years of age does not appear to be associated with substantial immunological interference for pooled serogroup B test strains. Although a lower immune response to some vaccine antigens in the MenABCWY group versus the 4CMenB group was observed, no firm conclusions can be made in relation to individual serogroup or strain comparisons between groups. The MenABCWY vaccine formulation had an acceptable reactogenicity profile, and no safety concerns were identified. These results support continued evaluation of this investigational vaccine.

## MATERIALS AND METHODS

### Study design.

This phase 2, randomized, open-label study was conducted at 16 centers in the Czech Republic between July and December 2018 (ClinicalTrials.gov Identifier NCT03587207). A study summary is available at www.gsk-studyregister.com (study identifier 208205). The study was conducted in accordance with the Declaration of Helsinki and Good Clinical Practice and was approved by the Multicentric Ethics Committee of Thomayer and IKEM Hospitals in Prague. All participants 18 years of age or older provided written informed consent, and parents or legally acceptable representatives provided written informed consent for participants younger than 18 years before enrollment. Anonymized individual subject data and study documents can be requested for further research from www.clinicalstudydatarequest.com.

The primary study objective was to assess the immune response to two doses of the investigational MenABCWY vaccine, two doses of the licensed 4CMenB and MenACWY vaccines administered concomitantly in the same arm or in different arms, and to a single dose of MenACWY vaccine at 1 month after the last vaccination. Immune responses 1 month post-first vaccine dose and safety and reactogenicity following vaccine administration were assessed as secondary objectives.

### Study participants.

A total of 500 adolescents and young adults 10 to 25 years of age were randomized to one of five study groups. Four groups received two vaccine doses 2 months apart (on study days 1 and 61) of MenABCWY, 4CMenB plus MenACWY administered concomitantly in the same arm, 4CMenB plus MenACWY administered concomitantly in different arms, or 4CMenB. The fifth group received one dose of MenACWY on day 1 of the study. Randomization was performed in a 1:1:1:1:1 ratio, with stratification by age group within each group (10 to 17 years and 18 to 25 years), according to a centrally held randomization list from the sponsor. Subjects were healthy and were excluded if they received any meningococcal vaccine, had a history of meningococcal disease, or had contact within 60 days of enrollment with an individual with meningococcal disease. Other exclusion criteria included immune system or blood disorders resulting from any cause or immune-mediated disease, known adverse reactions to vaccine components, receipt of immunoglobulins or blood products within 180 days before enrollment, receipt of an investigational product within 30 days before enrollment, any acute or chronic condition that could interfere with the results of the study, and a history of significant neurological disorder or seizure. Pregnant or breastfeeding women were excluded, and women of child-bearing potential had to commit to using birth control measures from 30 days before vaccination until 2 months after vaccination completion.

### Vaccines.

The MenABCWY investigational vaccine was prepared by reconstituting lyophilized powder containing CRM_197_-conjugated oligosaccharides of meningococcal serogroups A, C, W, and Y with a liquid suspension containing purified recombinant proteins from meningococcal serogroup B (NadA, fHbp, and NHBA) and outer membrane vesicle (OMV) components from serogroup B strain NZ98/254, as described previously (8). The reconstituted MenABCWY formulation contained 10 μg of MenA-CRM_197_, 5 μg each of MenC-CRM_197_, MenW-CRM_197_, and MenY-CRM_197_, 50 μg of each recombinant MenB protein, 1.5 mg aluminum hydroxide, and 25 μg of OMV. The MenACWY-CRM vaccine (Menveo) contained 10 μg of MenA-CRM_197_ and 5 μg each of MenC-CRM_197_, MenW-CRM_197_, and MenY-CRM_197_ and was prepared by mixing the lyophilized MenA-CRM_197_ component with the liquid MenCWY-CRM_197_ component just before injection. The 4CMenB vaccine (Bexsero), which contained 50 μg of each recombinant MenB protein, 1.5 mg aluminum hydroxide, and 25 μg of OMV, was a liquid suspension in a prefilled syringe. A 0.5-ml dose of each vaccine was administered intramuscularly into the deltoid muscle. For the 4CMenB+ACWY/S group, 4CMenB was injected in the upper deltoid and MenACWY in the lower deltoid of the nondominant arm. For the 4CMenB+ACWY/D group, 4CMenB was injected in the nondominant arm and MenACWY into the dominant arm.

### Immunogenicity analyses.

Blood samples were collected before and 1 month after each vaccine dose: i.e., on study days 1, 31, and 91 for all groups, apart from the MenACWY group, which provided blood samples on days 1 and 31. Antibody titers were measured by hSBA, using standardized procedures against a standard panel consisting of meningococcal serogroups A, C, W, and Y and serogroup B test strains, as described previously (8). The assay was performed by GSK laboratories, Marburg, Germany. Each of the serogroup B test strains measured bactericidal activity primarily directed against one of the major meningococcal antigens included in 4CMenB and the MenABCWY vaccine: strain M14459 for fHbp (factor H binding protein) variant 1.1, strain 96217 for NadA (neisserial adhesin A), strain NZ98/254 for major outer membrane protein antigen (PorA) P1.4, and strain M07-0241084 for NHBP (*Neisseria* heparin binding protein). Immune responses were expressed as hSBA GMTs, the percentage of participants with hSBA titers of ≥LLOQ, and the percentage with a 4-fold increase in hSBA titers. The LLOQ values for serogroup B test strains were 8 for fHbp, 8.6 for NadA, 8.2 for PorA, and 8.9 for NHBA and 22.7 for serogroup A, 5.2 for serogroup C, 39.6 for serogroup W, and 14.7 for serogroup Y. A 4-fold increase in hSBA titer postvaccination was defined as 4 times the limit of detection (LOD) or LLOQ (whichever is greater) for individuals with prevaccination titers below the LOD, 4 times the LLOQ for individuals with prevaccination titers between the LOD and LLOQ, or 4 times the prevaccination titer for individuals with prevaccination titers of ≥LLOQ. hSBA GMRs were calculated from GMTs against each serogroup 1 month after the last vaccination versus baseline (day 1).

### Safety analyses.

Participants were observed for 30 min after each vaccination for immediate reactions. Solicited local (erythema, swelling, induration, and pain at the injection site) and systemic (arthralgia, fatigue, nausea, headache, myalgia, and fever) AEs were reported by participants or parents/legally acceptable representatives on diary cards for 7 days following each vaccine dose. The severity of solicited AEs (apart from erythema, swelling, induration, nausea, and fever) was classified as mild, moderate (interfering with normal activity), or severe (preventing normal activity). Erythema, swelling, and induration were categorized as severe if over 100 mm in diameter. Severe nausea was defined as leading to minimal or no oral intake, and fever was defined as a body temperature of ≥38°C.

Unsolicited AEs were recorded for 30 days after each vaccine dose. SAEs, medically attended AEs, and AEs leading to withdrawal were reported over the entire study period, and the causal relationship of AEs to vaccination was assessed by investigators. The incidence of arthritis was assessed during the study as an AE of special interest.

### Statistical analyses.

This was a descriptive study, and no formal statistical hypothesis was tested. Approximately 100 subjects per vaccine group were to be enrolled. The immunogenicity analyses were conducted on the per-protocol population for immunogenicity, defined as all subjects who received any study vaccination and who had assay results available for at least one serogroup or serogroup B test strain at appropriate time points. An analysis of covariance (ANCOVA) model was fitted to pooled serogroup B test strains with study group, center, and strain as fixed effects and the log-transformed baseline titer with centering at zero as a continuous covariate. The percentage of subjects with titers above the LLOQ and associated 2-sided 80% Clopper-Pearson CIs (11) were computed by study group at baseline and 1 month after the last vaccination. In addition, differences in percentages of subjects with titers above the LLOQ and 2-sided 80% CIs between selected study groups were calculated using the method of Miettinen and Nurminen (12).

Potential immune interference due to immunological stress to lymph nodes was assumed to result in decreases in immune responses against all four serogroup B test strains, but to various extents. Assuming a standard deviation of 0.435 in log form, group sample sizes of 100 subjects in each treatment arm would achieve an approximate power of 80% to reject the null hypothesis of equal means when the population mean difference is −0.130 (or GMT ratio of 74%) with a standard deviation of 0.435 for both groups and a significance level (alpha) of 10% using a one-sided two-sample equal-variance *t* test. This calculation ignores potential correlation existing between serogroups of MenABCWY when pooled. When the four variants of serogroup B test strains are pooled, and using a 1-sided false-positive error rate of 10% with a power range of 80 to 90%, a global lymph node effect over GMT ratio of between 64% (or approximately −0.19 on a log_10_ scale) and 74% (−0.13 on log_10_ scale) can be detected. The 4CMenB+ACWY/S group was regarded as statistically inferior if the 2-sided 80% CI of the ratio of the GMT with 4CMenB+ACWY/D as control was lower than 1 at 1 month after the second vaccine dose. Although an 80% CI of the GMT ratio for MenABCWY versus 4CMenB+ACWY/S lower than 1 was taken as indicative of a possible difference between the groups, the study was not powered to detect any differences between groups by strain or serogroup. All subjects who received at least one study vaccination and provided safety data were included in the safety analyses, which were descriptive. Statistical analyses were performed using Statistical Analysis System (SAS) software version 9.3.
