# Supplementary figures and images for: Immunogenicity and Safety of Investigational MenABCWY Vaccine and of 4CMenB and MenACWY Vaccines Administered Concomitantly or Alone: a Phase 2 Randomized Study of Adolescents and Young Adults
Source: mSphere. 2021 Nov 17;6(6):e00553-21. doi: 10.1128/mSphere.00553-21 (PMC8597725; doi:10.1128/mSphere.00553-21)

A. Pooled serogroup B test strains


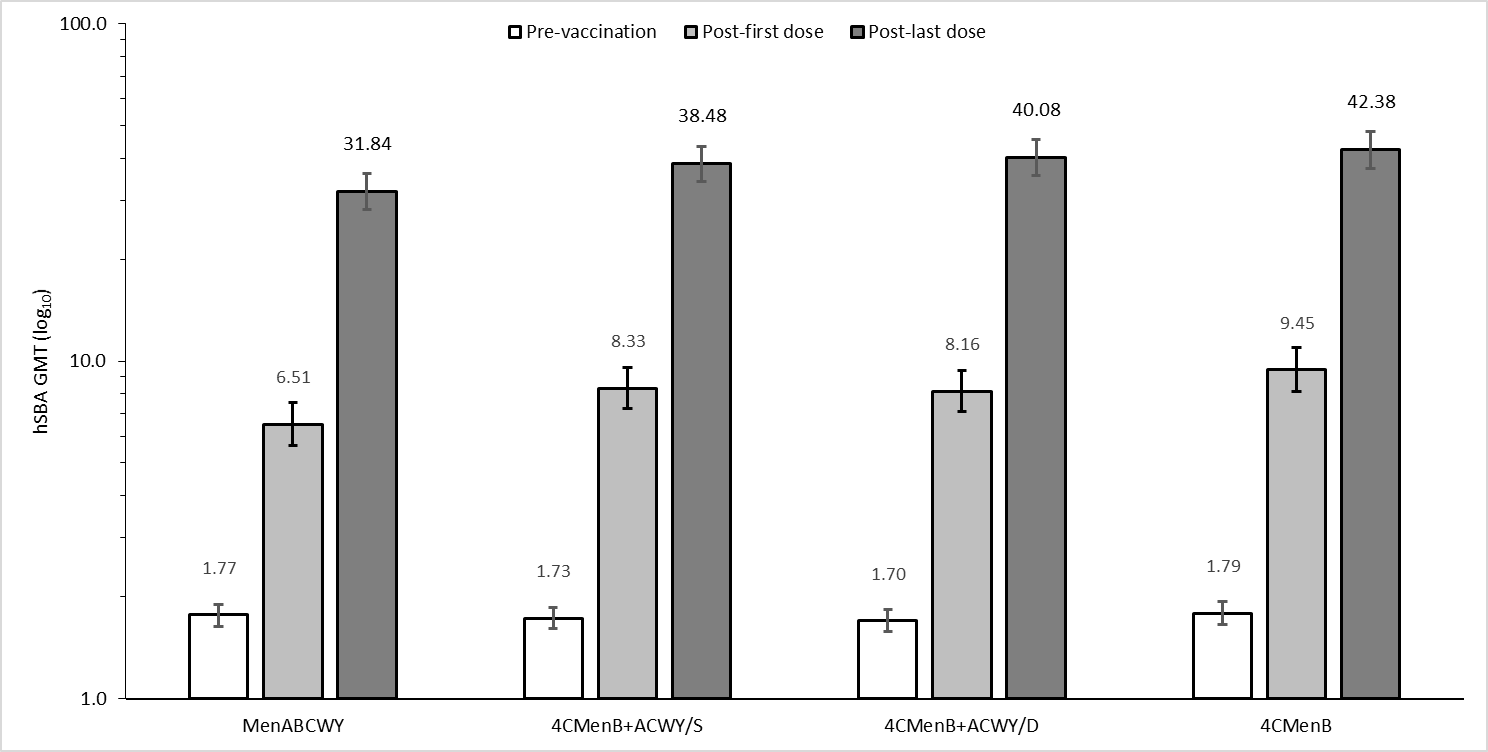


B. Serogroup B test strains


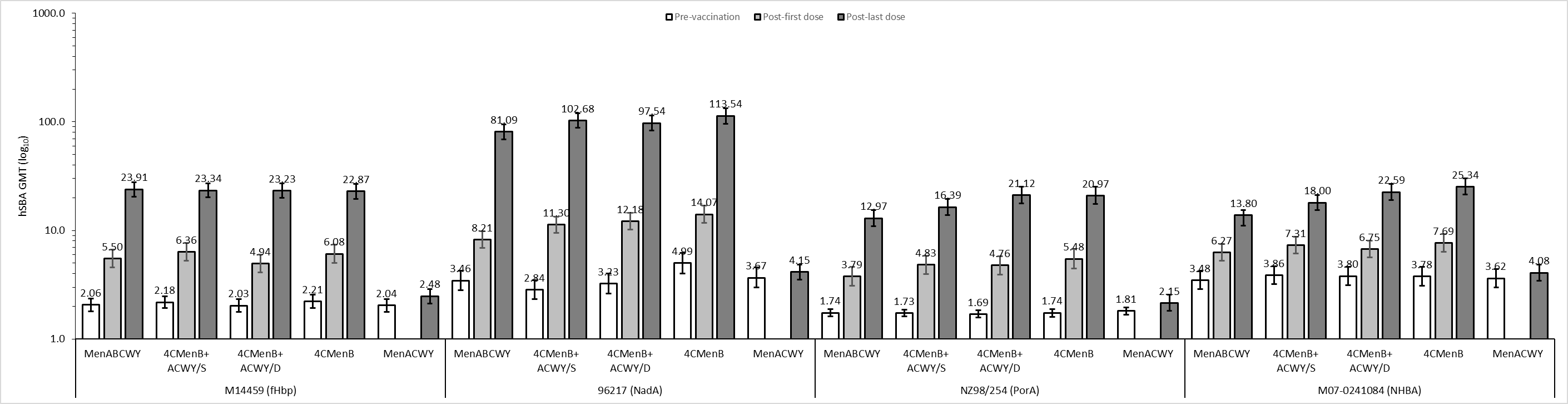


C. Serogroups A, C, W, and Y


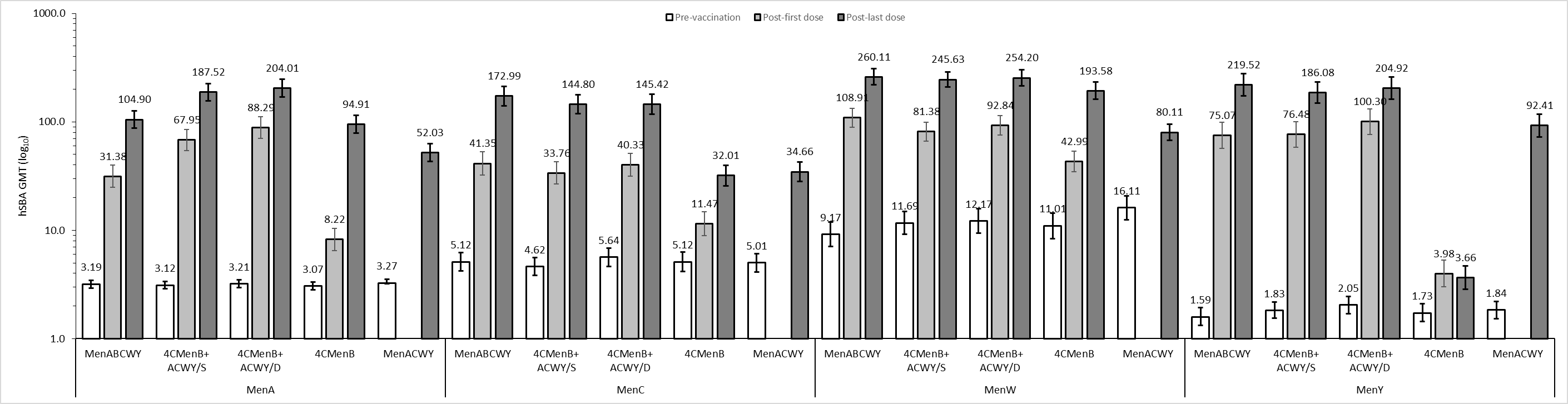

Supplement: FIG S1 [file msphere.00553-21-s0002.docx]

A. Serogroup B test strains


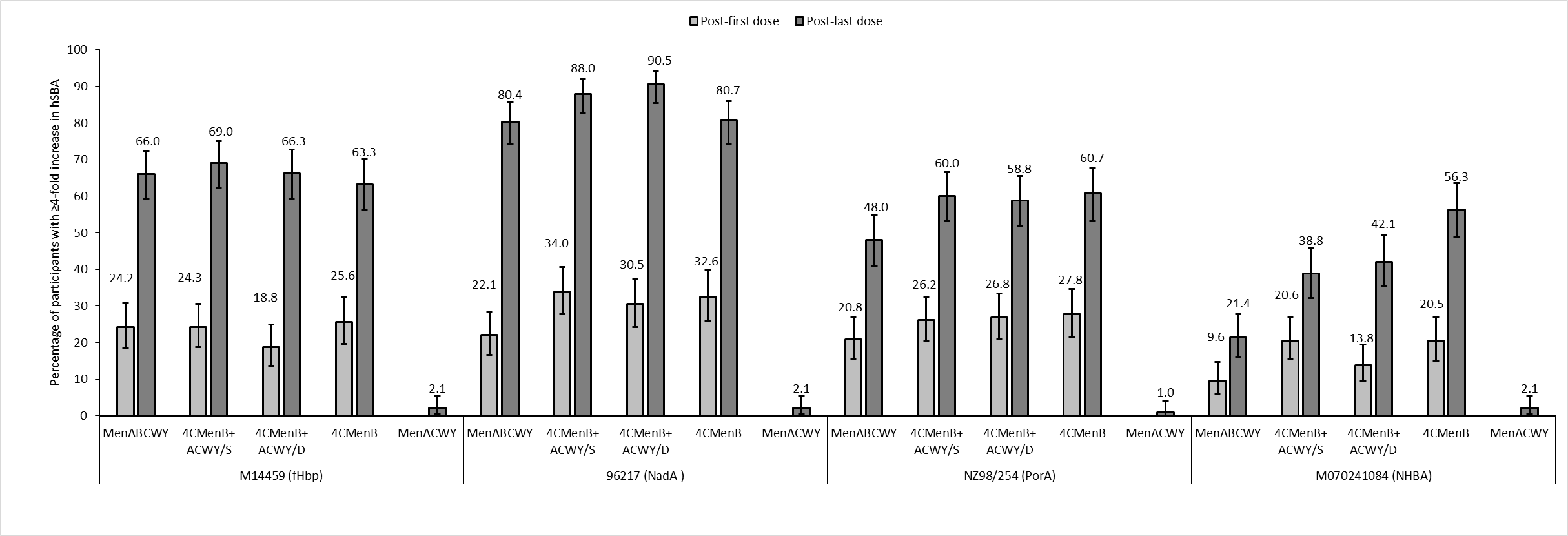


B. Serogroups A, C, W, and Y


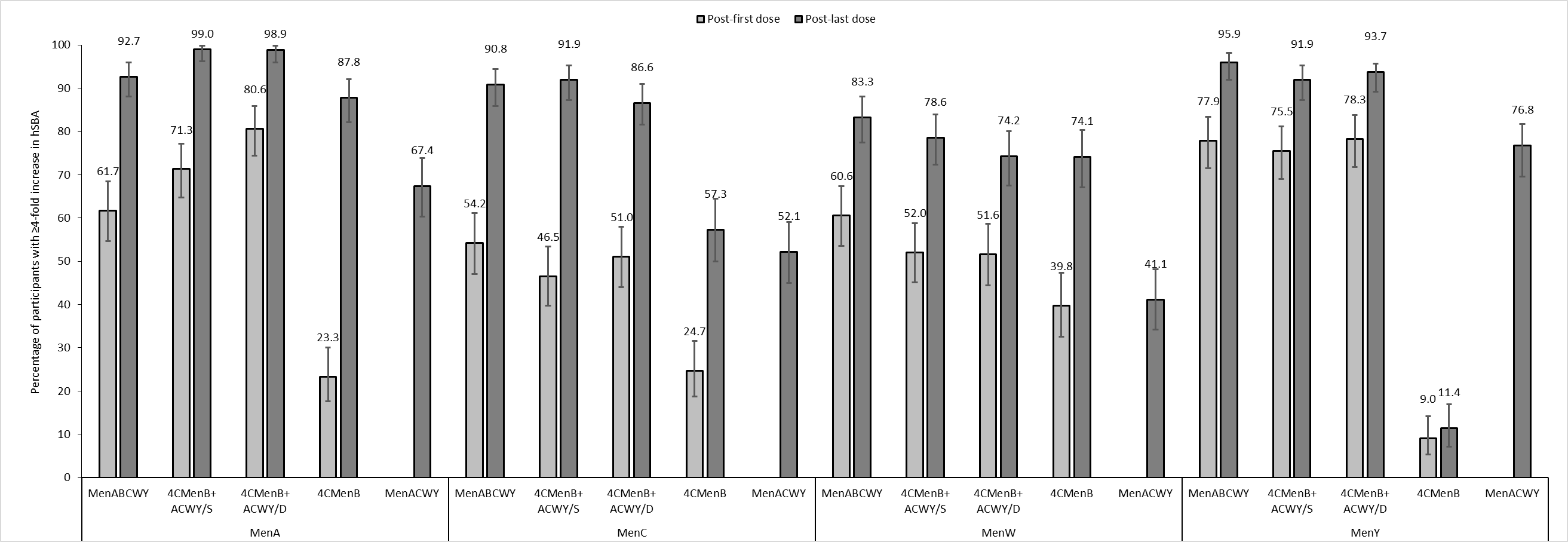

Supplement: FIG S2 [file msphere.00553-21-s0003.docx]

A. Serogroup B test strains


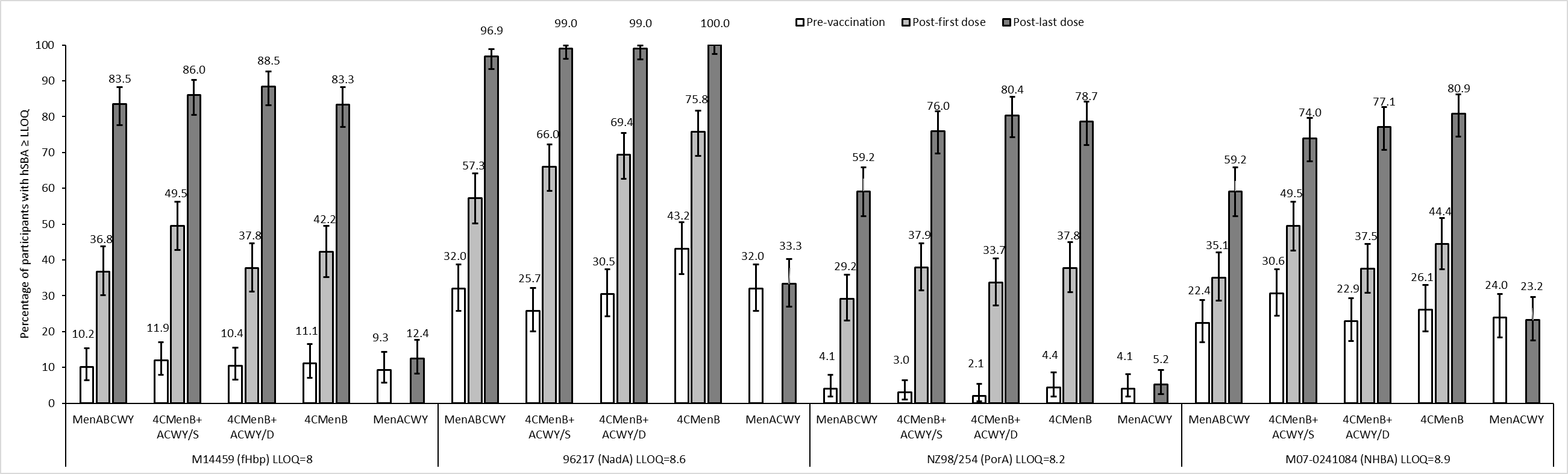


B. Serogroups A, C, W, and Y


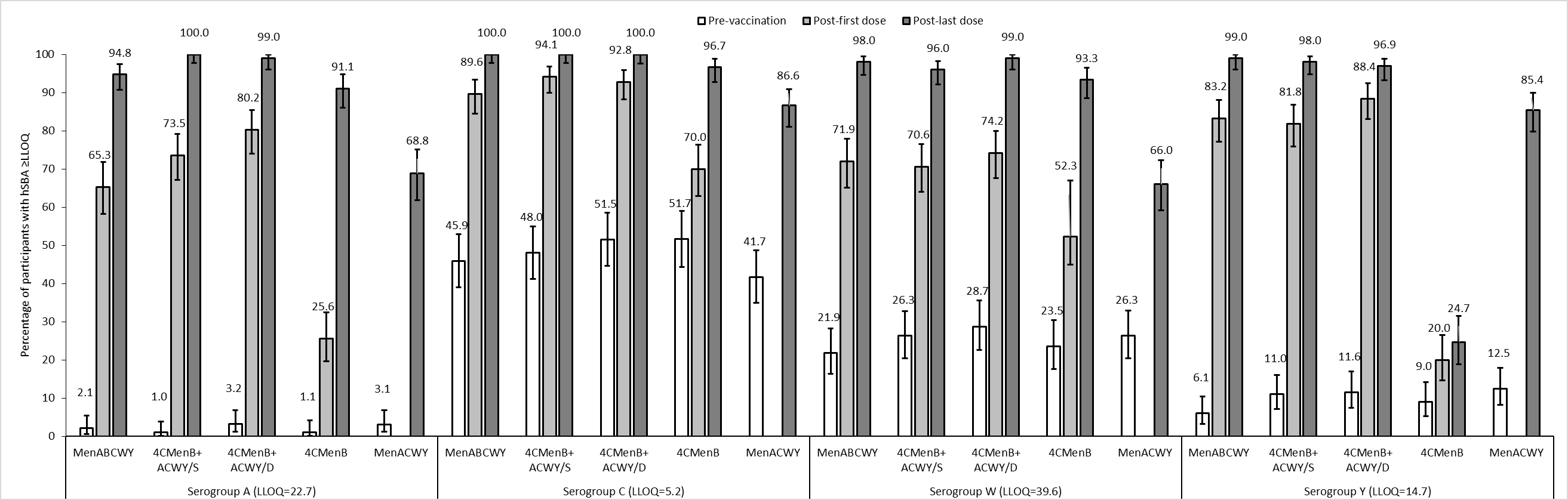

Supplement: FIG S3 [file msphere.00553-21-s0004.docx]
