# Supplementary material for: Immunogenicity and Safety of Investigational MenABCWY Vaccine and of 4CMenB and MenACWY Vaccines Administered Concomitantly or Alone: a Phase 2 Randomized Study of Adolescents and Young Adults
Source: mSphere. 2021 Nov 17;6(6):e00553-21. doi: 10.1128/mSphere.00553-21 (PMC8597725; doi:10.1128/mSphere.00553-21)
Supplement: TABLE S1 [file msphere.00553-21-s0001.docx]

|  | **Geometric mean ratio (80% CI)** | | | | |
| --- | --- | --- | --- | --- | --- |
|  | **MenABCWY (N=98)** | **4CMenB+ MenACWY/S (N=101)** | **4CMenB+ MenACWY/D (N=97)** | **4CMenB  (N=90)** | **MenACWY  (N=97)** |
| Serogroup B test strains |  |  |  |  |  |
| fHbp | 11.17 (9.59–13.02) | 10.90 (9.42–12.62) | 10.85 (9.30–12.66) | 10.69 (9.12–12.52) | 1.16 (0.99–1.35) |
| NadA | 21.33 (18.22–24.99) | 27.02 (23.22–31.44) | 25.66 (21.88–30.10) | 29.87 (25.32–35.24) | 1.09 (0.93–1.28) |
| PorA | 7.28 (6.11–8.67) | 9.20 (7.78–10.88) | 11.85 (9.94–14.13) | 11.77 (9.81–14.12) | 1.21 (1.01–1.44) |
| NHBA | 3.60 (3.05–4.25) | 4.96 (4.23–5.81) | 6.22 (5.27–7.35) | 6.98 (5.87–8.30) | 1.12 (0.95–1.33) |
| Serogroup A | 33.21 (27.55–40.04) | 59.36 (49.68–70.93) | 64.58 (53.52–77.93) | 30.05 (24.77–36.44) | 16.47 (13.62–19.93) |
| Serogroup C | 34.34 (27.88–42.30) | 28.74 (23.52–35.13) | 28.86 (23.40–35.60) | 6.35 (5.11–7.90) | 6.88 (5.58–8.49) |
| Serogroup W | 24.58 (20.72–29.15) | 23.21 (19.69–27.36) | 24.02 (20.20–28.56) | 18.29 (15.28–21.90) | 7.57 (6.37–8.99) |
| Serogroup Y | 106.87 (84.27–135.53) | 90.59 (72.18–113.70) | 99.76 (78.58–126.66) | 1.78 (1.39–2.28) | 44.99 (35.42–57.14) |
